# Fabrication and Evaluation of Nano-TiO_2_ Superhydrophobic Coating on Asphalt Pavement

**DOI:** 10.3390/ma14010211

**Published:** 2021-01-04

**Authors:** Hongfeng Li, Xiangwen Lin, Hongguang Wang

**Affiliations:** School of Civil Engineering, Northeast Forestry University, Harbin 150040, China; lihongfeng@nefu.edu.cn (H.L.); lxw1997@nefu.edu.cn (X.L.)

**Keywords:** superhydrophobic coating, nano-TiO_2_, asphalt pavement, water contact angle

## Abstract

In order to address water damage of asphalt pavement, reduce the occurrence of water-related potholes, deformation, and other diseases, and improve the performance and service life of the pavement, a nano-TiO_2_ superhydrophobic coating (PSC) on asphalt pavement was prepared from waterborne polyurethane and nano-TiO_2_ modified by stearic acid. FT-IR measured stearic acid successfully modified low surface energy substance on the surface of nano-TiO_2_. The SEM image shows that the PSC has a rough surface structure. The contact angle and rolling angle of the PSC in the contact angle test are 153.5° and 4.7°, respectively. PSC has a super-hydrophobic ability, which can improve the water stability of the asphalt mixture. Although the texture depth and pendulum value have been reduced by 2.5% and 4.4%, respectively, they all comply with the standard requirements. After the abrasion resistance test, the PSC coating still has a certain hydrophobic ability. These results surface PSC coating can effectively reduce water damage on asphalt pavement, and has considerable application value.

## 1. Introduction

The current research on asphalt pavement materials mainly focuses on the high and low- temperature performance and water stability of asphalt and asphalt mixtures [1]. In addition, some scholars have studied the road performance of asphalt mixtures through viscoelastic and viscoplastic models [2,3]. The water stability of asphalt pavements is mainly studied in this article, and we hope to eliminate the water damage of asphalt pavement through a superhydrophobic coating. Water damage is a very common problem on asphalt pavements. Under the influence of natural factors, rain and snow accumulate on the asphalt pavement surface. After a long period of erosion, the asphalt pavement will cause rutting, potholes, loose surfaces, net cracking, and other diseases [4]. The damage is large and wide, which will eventually reduce the service life of the asphalt pavement and increase the maintenance costs in the later period. There are roughly two research directions for preventing the early damage of asphalt pavement water damage: improving the waterproof and drainage capacity of the pavement, and improving the adhesion between the asphalt binder and the aggregate. Among them, improving the drainage and waterproof performance of the pavement structure is the most direct and effective measure [5], which can reduce the accumulation of water on the pavement and prevent water from entering the asphalt pavement structure to decrease the water damage.

The super-hydrophobic coating is a kind of coating with special surface wetting properties, its contact angle with water is greater than 150° and the rolling angle is less than 10° [6,7,8]. In 1997, W. Barthlott et al. studied the self-cleaning ability of plants and discovered for the first time that there are micro-nano-scale rough structures on the surface of the lotus leaf, and proposed the lotus leaf effect [9,10]. The super-hydrophobic surface has the excellent self-cleaning ability and can quickly remove surface water [11,12,13]. Methods of constructing superhydrophobic surfaces include the sol-gel method [14], vapor deposition method [15], the electrochemical method [16], the template method, etc. At present, scholars have carried out extensive research on the use of different nanoparticles for the biomimetic construction of superhydrophobic surfaces.

A long-term superhydrophobic self-cleaning coating was first fabricated by simply blending ambient-cured fluorinated polysiloxane binder with TiO_2_ nanoparticles [17]. The obtained coating has excellent durability in various environments. The water contact angle (WCA) of the coating is as high as 168.7° ± 2.4°, and the rolling angle is as low as 0.7° ± 0.3°. A PDMS/TiO_2_ composite superhydrophobic coating was successfully fabricated by blending PDMS solution with TiO_2_ nanoparticles [18]. The superhydrophobicity originates from the hierarchical structure of the different aggregation sizes of TiO_2_ nanoparticles within a robust crosslinked PDMS network, and PDMS coats the surface of TiO_2_ aggregates to offer low surface energy as well as high physical and chemical stability. Kim et al. [19] disperse PDMS-coated nano-TiO_2_ particles on the glass, showing a water contact angle close to 170°. With super-hydrophobicity, PDMS-coating was stable in the presence of electron beam and UV light, i.e., highly stable superhydrophobic surface based on TiO_2_ can be created by using PDMS-coating. Wang et al. [20] developed a simple and controllable method to fabricate a superhydrophobic coatings with improved transparency and thermostability through directly depositing hierarchical TiO_2_ micro/nanostructures on glass substrates, followed by thermal annealing and dipping modification with stearic acid. Qing et al. [21] report a simple and inexpensive method for fabricating fluorinated polysiloxane/ZnO nanocomposite coatings on steel substrates. The results showed that the ZnO nanoparticles were modified from hydrophilic to hydrophobic. When the weight ratio of modified-ZnO to fluorinated polysiloxane was 13:7, the contact angle of nanocomposite coating was 166°, and a sliding angle of 4°, was coating surface with hierarchical micro/nano-structures. Hua et al. [22] presented a simple method to fabricate superhydrophobic materials from TiO_2_ nanotube arrays (TNTAs). The hydrophilic TNTAs were functionalized with octadecyl phosphonic acid (ODPA) or 1H, 1H′, 2H, 2H′-perfluorodecyl phosphonic acid (PFDPA) to form a self-assembled monolayer on the TNTA surface to produce superhydrophobic ODPA@TNTA or PFDPA@TNTA surfaces. The superhydrophobic ODPA@TNTA and PFDPA@TNTA have contact angles of 156.0° ± 1.5° and 168° ± 1.5°, and contact angle hysteresis of 3.0° and 0.8°, respectively. The Zhou et al. [23] ambient-curable superhydrophobic fluoropolysiloxane/TiO_2_ nanocomposite coatings were prepared simply by blending a hydrophobic binder with TiO_2_ nanoparticles. The superhydrophobic coatings had good mechanical strength, excellent artificial weathering durability, and resistance to organic contaminants. A simple approach was proposed to fabricate a wear-resistant superhydrophobic coating by spraying the suspensions of polyurethane (PU)/molybdenum disulfide (MoS_2_) on various substrates. The PU/55.6%MoS2 coating with papillae-like rough structure showed superhydrophobic behavior with the water contact angle of 157° also has excellent wear resistance [24]. The above research shows that there are more mature methods for the construction of superhydrophobic surfaces, The research on applying it on the road is still in the initial stage.

In recent years, some scholars have begun to study the effect of superhydrophobic surfaces on pavement performance. The layer-by-layer (LBL) method was used to create an asphalt concrete surface coating with polytetrafluoroethylene (PTFE) as a well-known super-ice- and super-water-repellent material [25]. His other research is to synthesize, characterize, and evaluate the nanomaterials-based superhydrophobic (super water-repellent) coatings on Portland cement concrete (PCC) surfaces using layer-by-layer (LBL) deposition technique. Room-temperature vulcanized silicone rubber (RTV) is the main component of the superhydrophobic coating [26]. The micro-/nano-SiO_2_ particles modified by a silane coupling agent (KH550) were sprayed on the RTV surfaces to prepare the SC [27]. In another report, an acrylic superhydrophobic coating (ASC) on asphalt pavement was prepared from uncured acrylic acid and carbon nanotubes. The contact angle and rolling angle of water droplets on the ASC surface are 155.173° and 4.26°. The water permeability test results show that ASC can prevent water from penetrating the upper pavement structure, thereby reducing moisture damage. Photocatalytic, superhydrophobic, and self-cleaning capabilities were promoted on AC-6 and AC-14 asphalt mixtures by spraying of TiO_2_ and/or ZnO [28]. The contact angle test and surface morphology observation results indicate that the contact angle of water droplets increases effectively due to the rough micron-/nano- structures on the SC. By investigation into contact angles measured on the asphalt samples with different hydrophobic materials, the hydrophobic acrylate W4 made by Sigma-Aldrich Company (St. Louis, MO, USA) has the largest contact angle so that on average, contact angles have increased from 75° to 156° (the average angles has increased by 110%) and make the surface of the pavement super-hydrophobic [13].

In this paper, stearic acid is used to modify the surface of nano-TiO_2_ to improve the wettability and dispersion of the surface of nano-materials. The super-hydrophobic coating is prepared with modified nano-TiO_2_ and water-based polyurethane as the main materials. The hydrophobicity of nano-TiO_2_ superhydrophobic coating was measured by the contact angle test. We evaluated the physical, chemical, and morphological properties of the coating material by Fourier transform infrared spectroscopy (FT-IR) and scanning electron microscopy (SEM), and built a superhydrophobic coating on the surface of the asphalt mixture sample to study its water stability, anti-skid, and other performance.

## 2. Materials and Methods

### 2.1. Materials

Nano-TiO_2_ particles (10~20 nm in diameter, anatase phase) were purchased from Shanghai Jianghu Titanium White Chemical Co., Ltd., (Shanghai, China), and the specific surface area was 80~220 m^2^/g. Stearic acid was provided by Tianjin Zhiyuan Chemical Reagent Co., Ltd., (Tianjin, China). Ethanol was obtained from Xilong Science Co., Ltd., (Guangdong, China), all analytical grade. Waterborne polyurethane was purchased from Shenzhen Yoshida Chemical Co., Ltd., (Shenzhen, China). Asphalt binder and aggregate are provided by the construction site of Shuangbao Expressway, (Shuangyashan, China).

### 2.2. Preparation of Hydrophobic Nano-TiO_2_

Firstly, 5 g of nano-TiO_2_ was added to absolute ethanol (100 mL), stirred magnetically at room temperature for 15 min, and then ultrasonically dispersed for 30 min (80 W and 20 °C) to make it uniformly dispersed. Second, we weighed a certain mass of stearic acid (It is 20 wt.%, 60 wt.%, 80 wt.% of nano-TiO_2_ mass) and added it to the mixed solution of nano-TiO_2_ and absolute ethanol, magnetically stirred for 15 min, and then ultrasonicated for 30 min (80 W and 40 °C), and finally magnetically stirred for 2.5 h under certain temperature conditions (50 °C, 70 °C). After the mixed solution was left to stand for stratification, the supernatant was removed, dried in an oven at 80 °C, and the obtained powder was ground to obtain a hydrophobic nano-TiO_2_ particle. Table 1 shows the different preparation methods.

### 2.3. Fabrication of Superhydrophobic Coating for Contact Angle Test

**Figure 1:**
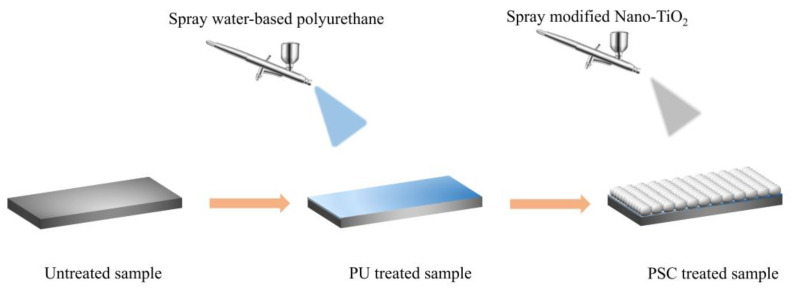
Schematic diagram of the preparation process.

First, 2.5 g of hydrophobic nano-TiO_2_ dispersed in 50 mL of absolute ethanol and magnetically stirred for 30 min at ambient temperature. At the same time, 3 g of water-based polyurethane is uniformly dispersed into an appropriate amount of ethanol solution. Washed with anhydrous ethanol 7.62 cm × 2.54 cm slide glass and dried for future use. We used a spray gun to spray a layer of aqueous polyurethane dispersion on a glass slide. Subsequently, the nano-TiO_2_ dispersion was sprayed onto the surface of the uncured polyurethane, which was dried for 1 h in an oven at 80 °C in order to form a cured superhydrophobic coating (PSC). When used for asphalt mixture samples, we chose room temperature curing. The distance between the airbrush and substrates was about 20 cm. A schematic diagram of the preparation process is shown in Figure 1.

### 2.4. Preparation of Asphalt Mixture

**Figure 2:**
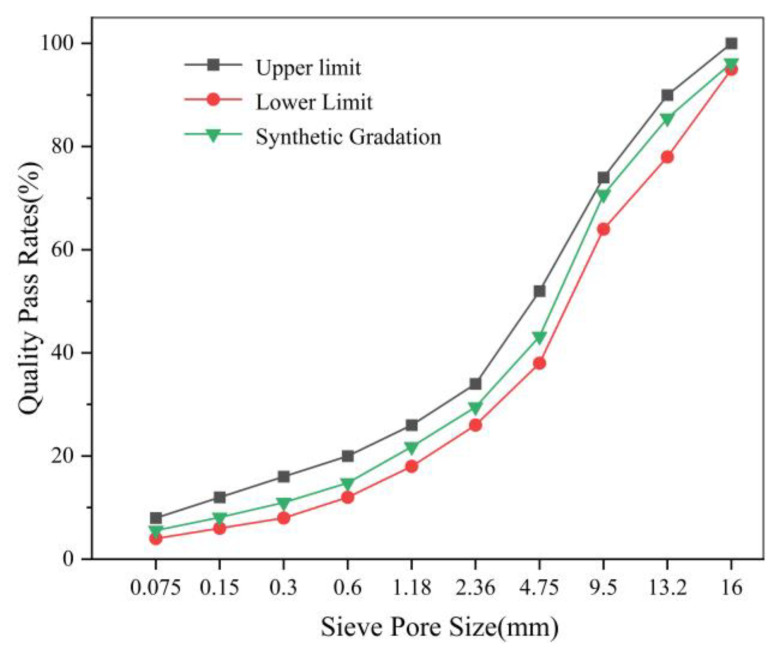
AC-16 asphalt mixture gradation design.

According to the standard molded AC-16 asphalt mixture Sample in the Chinese Standard Test Methods of Bitumen and Bituminous Mixtures for Highway Engineering (JTG E20-2011) [29], AC-16 asphalt mixture gradation design is shown in Figure 2.

### 2.5. Water Contact Angle (WCA) Test

The contact angles and rolling angles of the hydrophobic nano-TiO_2_ and the superhydrophobic coating were all measured using an optical contact angle tester (OCA20, DataPhysics Instruments, Filderstadt, Germany) [30]. Among them, the hydrophobic nano-TiO_2_ is also sprayed on the glass slide. The measurement is performed by the static drop method, and the syringe is controlled to drop 5 μL of water on the test sample, and the contact angle can be obtained by fitting with the supporting software.

### 2.6. Characterization

In addition to measuring the water contact angle and rolling angle of the coating material to characterize its surface wettability, FT-IR and SEM characterization methods were also used to analyze the reaction mechanism of stearic acid modified nano-dioxide and the micro-morphology of the coating surface.

Elemental composition and chemical groups of PSC samples were recorded by a Fourier transform infrared spectrometer (FT-IR, Spectrum 400, Perkin Elmer Instruments, Akron, Ohio, USA) to analyze the changes of surface chemical functional groups before and after the modification of nano-TiO_2_ by stearic acid. The stearic acid and nano-TiO_2_ powder before and after modification were tested, respectively. The sample was made by the tableting method, and KBr powder was used as the diluent because the solid sample cannot be directly used for tableting. The test samples consisted of 2 mg of powder and 200 mg of KBr powder.

Surface micro-morphology of PSC samples was observed by a field emission scanning electron microscope (FESEM, JSM-7500F, JEOL, Tokyo, Japan). All test samples were sprayed on the glass slide and cured before spraying gold to enhance its conductivity.

### 2.7. Evaluation of Water Stability of Asphalt Mixture

#### 2.7.1. Water Absorption Test

According to the standard T0705-2011 in the Chinese Standard Test Method of Bitumen and Bituminous Mixtures for Highway Engineering (JTG E20-2011) [29], the water absorption rate of the asphalt mixture sample was measured through an overflow tank.

#### 2.7.2. Water Permeability Test

We measured the volumes of water permeated according to the T0730-2011 Asphalt Mixture Water Permeability Test Method in the Chinese Standard Test Method of Bitumen and Bituminous Mixtures for Highway Engineering (JTG E20-2011) [29] to evaluate the effect of superhydrophobic coating on the water permeability coefficient of asphalt mixture samples.

### 2.8. Anti-Skid Performance Test of Asphalt Pavement

The coating material affects the surface roughness of the asphalt pavement and its anti-skid performance. The anti-skid performance of the asphalt pavement is very important to road traffic safety. Two indexes of texture depth and pendulum value were used to measure the influence of coating materials on the anti-skid performance of asphalt pavement.

#### 2.8.1. Surface Texture Depth Test

According to the standard T0731-2000 in the Chinese Standard Test Method of Bitumen and Bituminous Mixtures for Highway Engineering (JTG E20-2011) [29], the paving area of homogeneous sand on the surface of the sample was tested to calculate the texture depth.

#### 2.8.2. British Pendulum Number Test

The pendulum number was measured according to the T0964-2008 Test Method in the Chinese Field Test Regulations for Highway Subgrade and Pavement (JTG 3450-2019) [31]. During the test, the ambient temperature was adjusted to 20 °C to facilitate data processing.

### 2.9. Wet Track Abrasion Test

**Figure 3:**
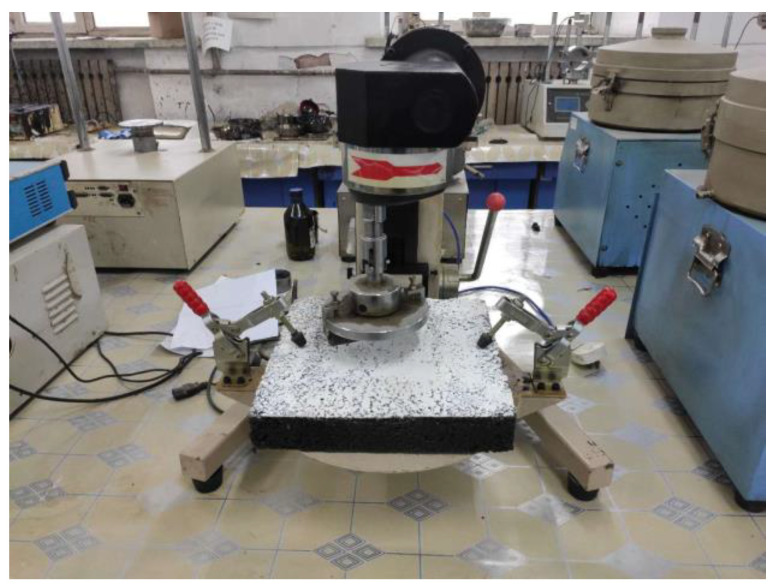
Wet track abrasion test.

According to the standard T0752-2011 in the Chinese Standard Test Method of Bitumen and Bituminous Mixtures for Highway Engineering (JTG E20-2011) [29], wet track abrasion meter was used to simulate the consumption of the coating on the wheel rotation. The test is shown in Figure 3.

## 3. Results and Discussions

### 3.1. Water Contact Angle and Rolling Angle Test

**Figure 4:**
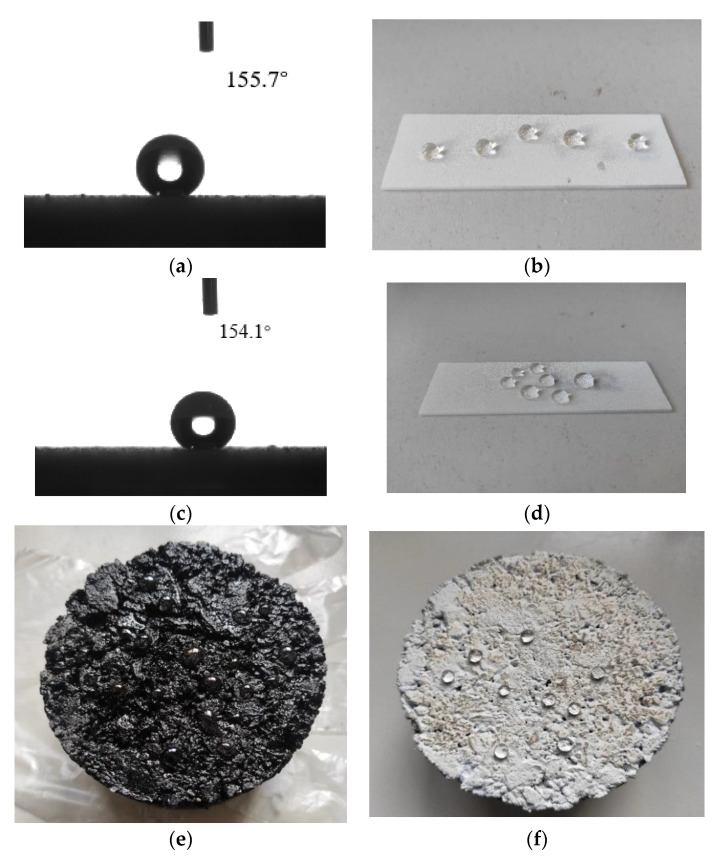
Image of the water contact angle of the sample: (**a**) Contact angle testing picture of S1; (**b**) photo of actual item contact angle of S1; (**c**) contact angle testing picture of cured superhydrophobic coating (PSC); (**d**) photo of actual item contact angle of PSC; (**e**) contact angle of untreated asphalt Marshall specimen; (**f**) contact angle of PSC asphalt Marshall specimen.

The contact angle test alone cannot evaluate the dynamic stability of surface wettability. Generally, the contact angle and the rolling angle are combined to evaluate the superhydrophobic ability of the material surface [32]. The test results are shown in Table 2 and Figure 4. It can be concluded that the water contact angle of nano-TiO_2_ modified by stearic acid is 154.8° and the rolling angle is 2.9°, successfully changed the surface wettability of nano-TiO_2_ from hydrophilic to superhydrophobic. By comparison, it can be concluded that the modification method of S1 sample is the best. The water contact angle of PSC is 153.5°, and the rolling angle is 4.7°. This means that can make the asphalt pavement have superhydrophobic ability.

The test results show that by a simple modification of stearic acid, the water contact angle of nano-TiO_2_ can be increased to 154.8°. After waterborne polyurethane and nano-titanium dioxide work together the water contact angle will slightly decrease. The analysis is because a part of the nano-titanium dioxide is encapsulated by polyurethane, but it can still obtain super-hydrophobicity from the surface of the sample. This means that spraying the PSC coating, which can be seen in Figure 4e,f, can significantly increase the water contact angle of the asphalt pavement surface. At the same time, the lower rolling angle can drain the surface water of the road through the drainage ditches quickly. It can effectively reduce the time of water residing on the road surface, thereby reducing water damage.

### 3.2. Characterization of As-Prepared Samples

**Figure 5:**
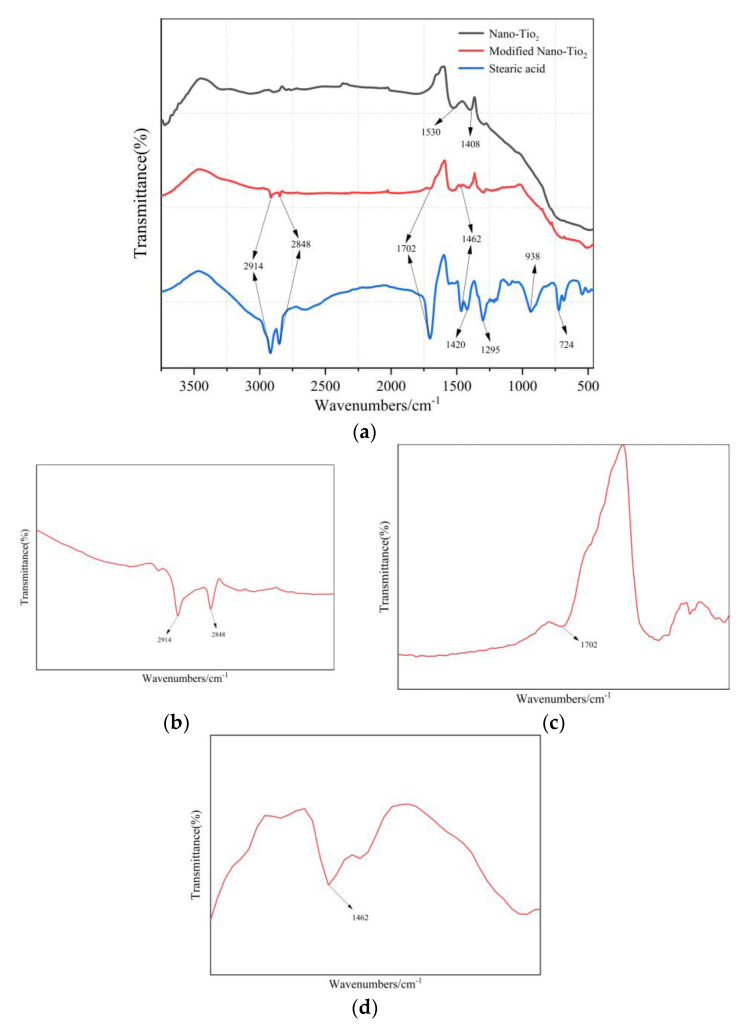
Fourier transform infrared spectroscopy curves: (**a**) Fourier transform infrared spectrum curves of stearic acid and nano-TiO_2_ before and after modification; (**b**–**d**) a magnified view of the wavenumber range of characteristic peaks.

**Figure 6:**
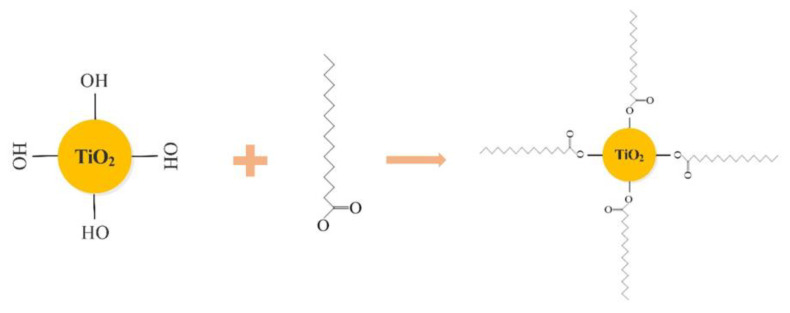
Schematic of stearic acid interaction with TiO_2_.

FT-IR spectrum test results are shown in Figure 5. Among them, Figure 5b shows that the peaks located at 2848 cm^−1^ and 2914 cm^−1^ in the IR spectrum, the analysis reason is caused by the symmetrical and antisymmetric stretching vibration of CH_2_ and CH_3_ [33,34]. The peak at 1702 cm^−1^ in Figure 5c is attributed to the stretching vibration of C=O in the COOH group [35]. The absorption peak at 1462 cm^−1^ corresponds to the deformation vibration of CH_2_ and CH_3_ groups (Figure 5d) [36]. These peaks were not found in the IR spectrum of nano-TiO_2_, indicating that stearic acid has successfully modified nano-TiO_2_, especially the introduction of CH_3_ and CH_2_ groups that can reduce its surface energy. Stearic acid is composed of non-polar hydrophobic alkane long chain and hydrophilic carboxyl groups. When stearic acid modifies nano-TiO_2_, the COOH in stearic acid reacts with the -OH group of TiO_2_ [37]. Their reaction mechanism is shown in Figure 6.

**Figure 7:**
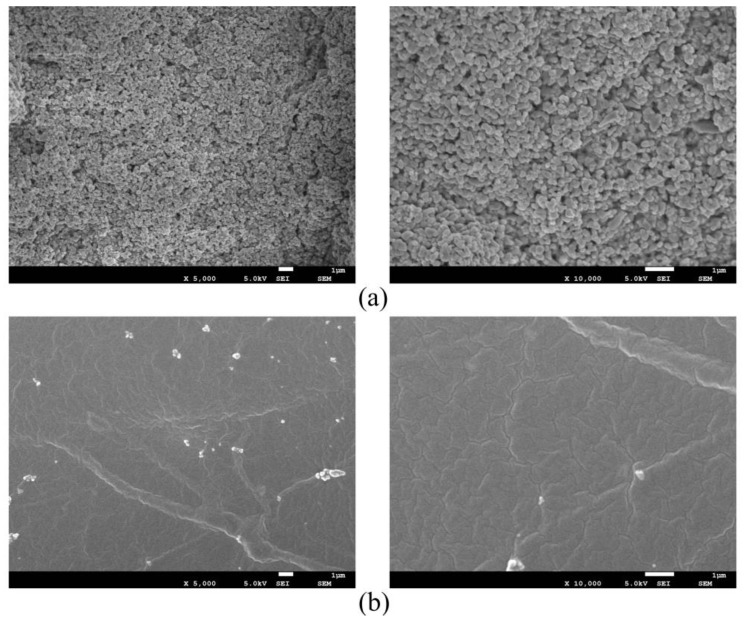
SEM test results: (**a**) SEM photo of PSC coating surface; (**b**) SEM photo of the waterborne polyurethane coating surface.

The surface morphology of pure waterborne polyurethane coating and PSC coating is shown in Figure 7, which can be seen from the SEM image with different magnifications that the surface of the pure water-based polyurethane coating is relatively smooth, and the corresponding contact angle test result is 108.0°. The surface of the PSC coating has a certain degree of roughness, and it can be observed that the nanoparticles are uniformly stacked on the surface, and the contact angle is also increased to 153.5°. The reason for the analysis is that the nano-TiO_2_ modified by stearic acid forms a micro-nano binary rough structure on the surface of the coating, which makes the coating obtain a super-hydrophobic effect.

Two key factors for constructing superhydrophobic surfaces are low surface energy and surface roughness [38,39]. The results of the microscopic test show that stearic acid introduced CH_2_ and CH_3_ groups in this study to reduce nano-TiO_2_ surface energy, and modified nano-TiO_2_ formed micro- and nano-scaled rough structures on the surface of the sample. Micro/nanometer-scale particles, and low surface energy CH_2_ and CH_3_ groups render the PSC coating superhydrophobic. Based on the PU hydrophobic coating, the two form a dual synergistic effect, which is the fundamental reason for a coating to obtain the super-hydrophobic ability.

### 3.3. Water Stability Test Results

**Figure 8:**
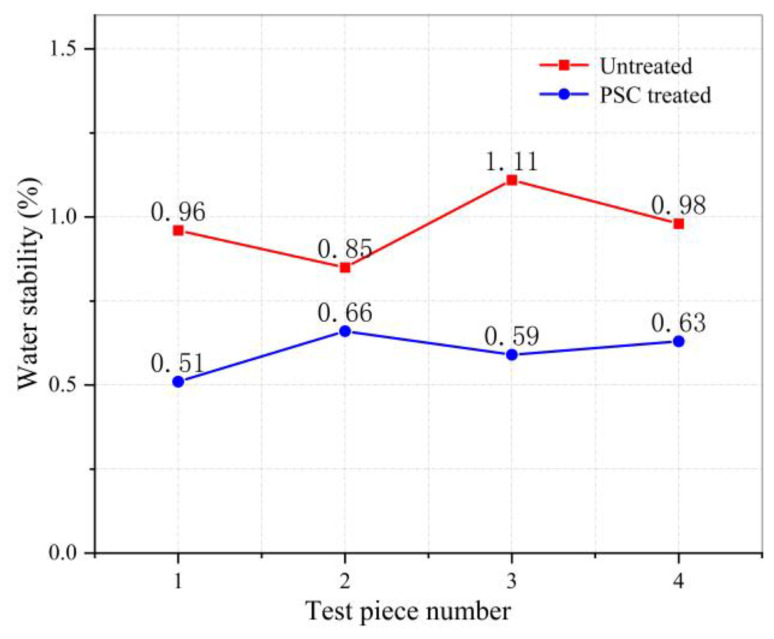
Point line graph of water absorption test results.

In the water absorption experiment, first, the mass of the dry specimen was weighed in the air (m_a_), the overflow tank is maintained at a temperature (25 ± 0.5) °C and placed the specimen in a basket soaked in water for 3~5 min, and the mass of specimen was weighed in the water (m_w_). Then, remove the test piece from the water, use a clean and soft wrung-out wet towel to gently wipe off the surface water of the test piece, and weigh the surface dry mass (m_f_) of the test piece. The water absorption (S_a_) of the asphalt mixture test piece can be calculated according to Equation (1). The test results are expressed in Table 3 and Figure 8.

(1) $$S_{a}=\frac{m_{f}-m_{a}}{m_{f}-m_{w}}\times 100\%$$

In the water permeability (*C*_w_) test, the untreated asphalt mixture sample is first tested, after the test is completed, it is allowed to dry naturally, use PSC to treat it and measure again. When the water surface drops to the 100 mL mark, immediately start the stopwatch to start timing. Every 60 s, record the scale of the instrument tube until the water surface drops 500 mL. When the water surface drops slowly, the water seepage volume can be stopped after measuring 3 min; if the water surface drops to a certain level, it basically stays still, indicating that it is impervious. The water permeability coefficient of the asphalt mixture test piece can be calculated according to Equation (2). The test results are expressed in Table 4.

(2) $$C_{w}=\frac{V_{2}-V_{1}}{t_{2}-t_{1}}\times 60$$

In Equation (2), *V_1_* and *V_2_* are the reading of the graduated cylinder scale during the first and second timing respectively, and the unit is mL. *t_1_* and *t_2_* are the time of the first and second timing respectively, the unit is second.

Through the water absorption and water permeability test results, it can be concluded that the PSC treated asphalt mixture samples have a significant reduction in water absorption, which is 60.5% lower than that of the untreated samples, and the surface of the PSC asphalt mixture sample is impermeable. This shows that PSC can form a water-repellent sealing layer on the asphalt pavement surface while reducing the time that water stays on the surface of the pavement and preventing water from entering the pavement, thereby avoiding damage to the adhesion between asphalt binder and aggregate to achieve the purpose of preventing water damage. For asphalt pavements, the inability of water to enter will naturally reduce the probability of water damage, which is of great significance to prevent the early damage of asphalt pavement water damage.

### 3.4. Anti-Skid Test Results

**Figure 9:**
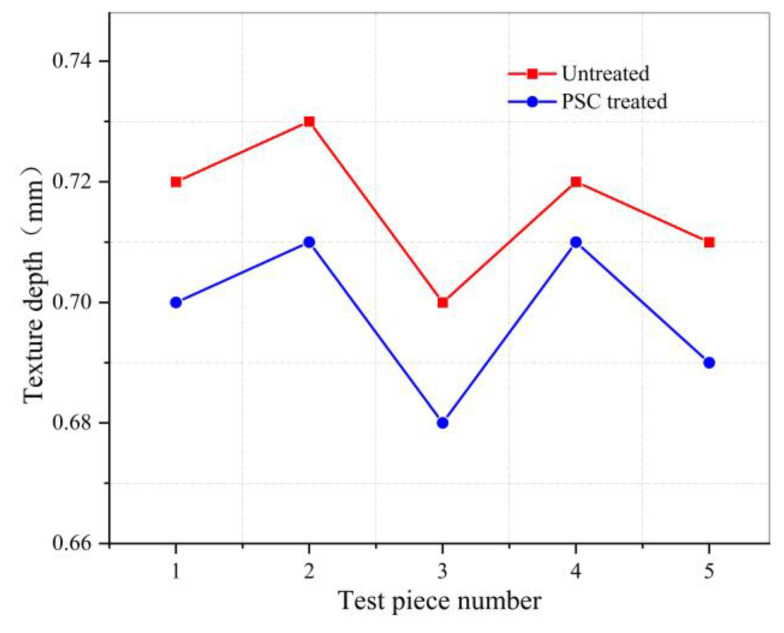
Point line graph of texture depth test results.

The sand was poured onto the surface of the asphalt mixture sample, we spread the sand into a circle with a push plate, and do not leave floating sand on the surface. The diameter of the two perpendicular directions of the circle formed by a steel ruler measure, and we take the average value. The texture depth (*MTD*) is calculated by Equation (3). After the test is completed, surface of the untreated sample is cleaned and PSC is sprayed to continue the test, the results are shown in Table 5 and Figure 9.

(3) $$MTD=\frac{1000V}{\pi D^{2}/4}$$

In Equation (3), V is the volume of sand (25 cm^3^), *D* is the average diameter of the sand after spreading out, and the unit is mm.

**Figure 10:**
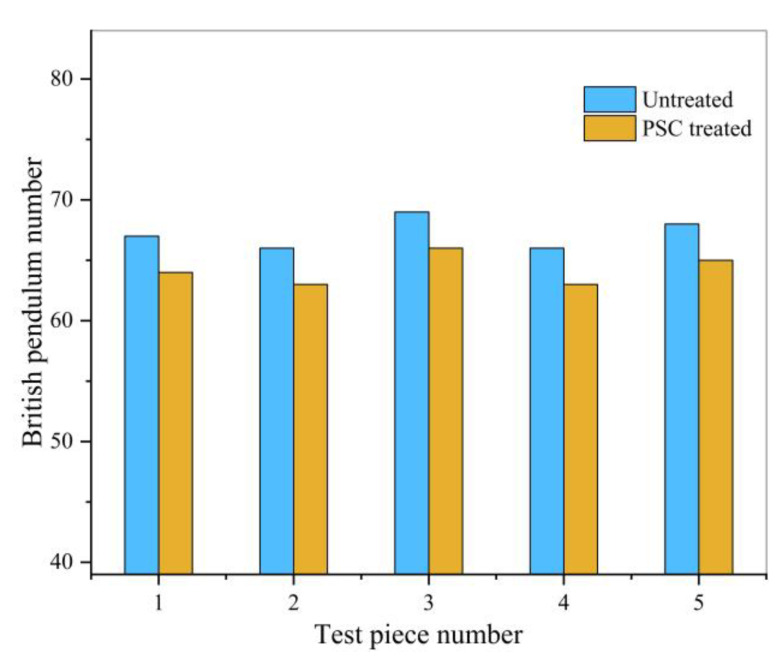
Pendulum number test results.

In the pendulum number test, a portable pendulum instrument was used to determine the friction coefficient of the asphalt mixture sample, measure the height of the backswing, and evaluate the friction resistance of the sample surface. The untreated and PSC treated samples were tested separately. The results are shown in Table 6 and Figure 10.

It can be seen from the anti-skid test results that the texture depth and pendulum number of the sample treated with PSC will be reduced. After spraying PSC, MTD is reduced by an average of 2.5%, and British Pendulum Number (BPN) is reduced by an average of 4.4%, but the amplitude is small and meets the standard requirements. This shows that PSC will fill the texture of the asphalt pavement surface and affect the anti-skid performance of the pavement. It will not affect traffic safety, which is the primary premise that PSC can be applied to asphalt pavement.

### 3.5. Coating the Abrasion Resistance Test

**Figure 11:**
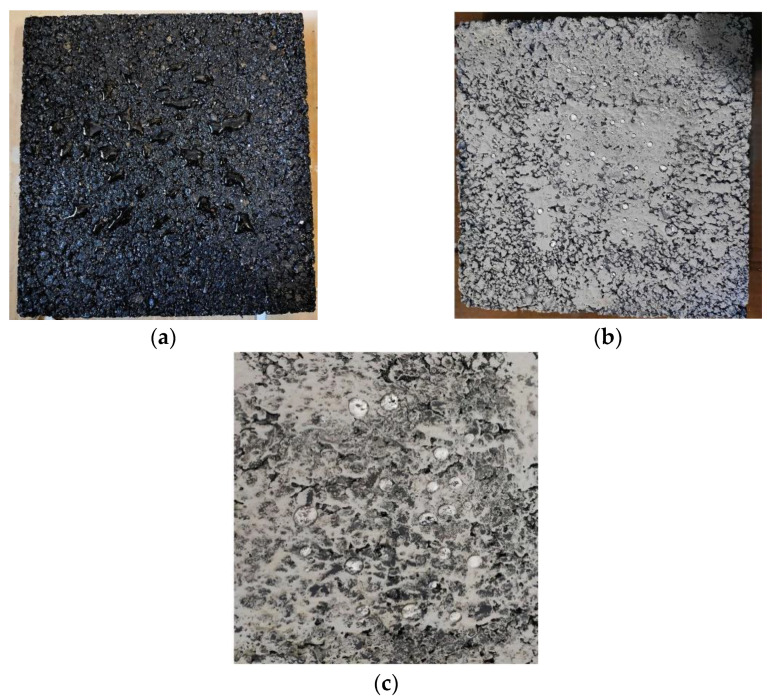
Sample water contact state before and after abrasion: (**a**) Water contact state of untreated sample surface; (**b**) water contact state of PSC treated sample surface; (**c**) the water contact state of the sample surface at the abrasion position.

Since the super-hydrophobic coating layer is directly exposed to the external environment, under the action of automobile tires, it is easy to get wear damage and reduce the super-hydrophobic performance. Therefore, refer to the wet track abrasion test in the standard to conduct abrasion test on PSC treated asphalt mixture samples, and the time is 30 min. The water contact state of the asphalt mixture was observed before and after abrasion. The result is shown in Figure 11.

Through the wet track abrasion test to simulate the loss of the coating caused by the driving of the vehicle, after 30 min of wear, the wear surface still has a certain hydrophobic effect. The test results show that PSC will be filled into the surface texture of the asphalt pavement, the wheels will cause less damage to the coating. The coating has a certain degree of durability, which also provides reliable support for its wide application.

## 4. Conclusions

In this paper, the super-hydrophobic coating PSC was prepared mainly using waterborne polyurethane and modified nano-TiO_2_. Study the modification mechanism of nano-TiO_2_ and the microstructure of the coating surface. Comprehensive evaluation of the water stability, and Anti-skid of PSC asphalt mixture samples. At the same time, the abrasion resistance of the coating was also studied. Through these to reflect the effect of PSC on asphalt pavement, the following conclusions could be drawn:

(1) The water contact angle and rolling angle of PSC are 153.5° and 4.7°, respectively. It can make the surface of the asphalt pavement obtain superhydrophobic ability, so that the water could be discharged from the surface of the pavement quickly.
(2) Based on FT-IR and SEM. It could be seen that the low surface energy modification of stearic acid transforms nano-TiO_2_ from hydrophilic to super-hydrophobic, and PSC has successfully constructed a micro-nano rough structure on the surface of the asphalt mixture specimen.
(3) The water absorption and water permeability test results show PSC can improve the water stability of the asphalt mixture and form an impervious layer on the surface of the road to prevent early water damage.
(4) Although PSC will have a certain negative impact on the anti-skid performance of asphalt pavement, the impact is small—all meet the requirements of use.
(5) The coating abrasion resistance test shows that PSC has a certain degree of abrasion resistance on asphalt pavement, and it is not easy to cause loss under wheel friction.

In this study, a spraying method was used to construct a superhydrophobic surface with modified nano-TiO_2_ and water-based polyurethane as the main materials. Contrast with sol-gel method, vapor deposition method, electrochemical method, template method, etc. The coating has a simple preparation process, low cost, excellent superhydrophobic properties, and some wear resistance. Test results show that it can effectively improve the ability of the road surface to withstand water damage and increase its service life. The coating is expected to be used in actual engineering.

## Figures and Tables

**Table 1:** Preparation method of hydrophobic nano-TiO_2_.

Groups of Nano-TiO_2_ Sample	Stearic Acid Content (g)	Whether Ultrasonic Dispersion	Final Stirring Temperature (°C)
S1	3	Yes	70
S2	3	Yes	50
S3	1	Yes	70
S4	4	Yes	70
S5	3	No	50
S6	3	No	70

**Table 2:** Water contact angle and rolling angle test results.

Sample Serial Number	Measured Contact Angle (°)			Average Value of Measured Contact Angle (°)	Maximum Angle (°)	Variance
	Test Point 1	Test Point 2	Test Point 3			
S1	154.6	154.1	155.7	154.8	155.7	0.45
S2	153.9	148.7	153.7	152.1	153.9	5.79
S3	151.8	154.5	152.3	152.9	154.5	1.38
S4	150.1	149.3	150.4	149.9	150.4	0.22
S5	152.9	150.1	153.2	152.1	153.2	1.95
S6	149.8	154.3	152.7	152.3	154.3	3.47
PU	107.8	109.9	106.2	108.0	109.9	2.30
PSC	153.8	154.1	152.7	153.5	154.1	0.36
	**Measured rolling angle (°)**					
S1	2.7	2.5	3.5	2.9	3.5	0.19
PU	>10	>10	>10	>10	--	--
PSC	4.3	4.7	5.0	4.7	5	0.08

**Table 3:** Water absorption test results.

Sample Group	m_a_ (g)	m_w_ (g)	m_f_ (g)	S_a_ (%)	Average Value (%)	Reduced Ratio (%)
PSC treated	1183.3	695.5	1185.8	0.51	0.60	60.5
	1179.7	699.5	1182.9	0.66		
	1181.9	692.9	1184.8	0.59		
	1184.8	694.3	1187.9	0.63		
Untreated	1184.5	700.6	1189.2	0.96	0.98	
	1181.0	700.8	1185.1	0.85		
	1183.0	702.3	1188.4	1.11		
	1176.7	700.7	1181.4	0.98		

**Table 4:** Water permeability test results.

Sample	Water Permeability Coefficient (mL/min)	Average Value (mL/min)
Untreated	26.6	27.5
	28.1	
	27.9	
PSC treated	impermeable	impermeable
	impermeable	
	impermeable	

**Table 5:** Texture depth test results.

MTD of Untreated Samples (mm)	MTD of PSC Treated Samples (mm)	Reduced Ratio (%)	Average Reduced Ratio (%)	Standard Requirement (mm)
0.72	0.70	2.8	2.5	≥0.55
0.73	0.71	2.7		
0.70	0.68	2.9		
0.72	0.71	1.4		
0.71	0.69	2.8		

**Table 6:** Pendulum number test results.

Sample	British Pendulum Number					Average Number	Reduce the Magnitude (%)	Standard Requirement
Untreated	67	66	69	66	68	67.2	4.4	≥45
PSC Treated	64	63	66	63	65	64.2		

## Data Availability

No new data were created or analyzed in this study. Data sharing is not applicable to this article.
